# Cloprostenol sodium improves reproductive performance of multiparous sows during lactation

**DOI:** 10.3389/fvets.2024.1342930

**Published:** 2024-01-31

**Authors:** Xuedan Zhu, Xinke Zhang, Yuqing Zhang, Jiahao Li, Siqi Li, Siqi Zhang, Li Li, Li Meng, Hengxi Wei, Shouquan Zhang

**Affiliations:** State Key Laboratory of Swine and Poultry Breeding Industry, National Engineering Research Center for Breeding Swine Industry, Guangdong Provincial Key Lab of Agro-animal Genomics and Molecular Breeding, College of Animal Science of South China Agricultural University, Guangzhou, China

**Keywords:** *D*-cloprostenol sodium, *DL*-cloprostenol sodium, reproductive performance, milk performance, farrowing induction, multiparous sow

## Abstract

This study aimed to determine the effect of prostaglandin F_2α_ (PGF_2α_) analog (*D*-cloprostenol sodium and *DL*-cloprostenol sodium) administration on the milk yield of multiparous sows (MS) and piglet growth performance. In total, 320 Landrace×Yorkshire parturient MS were randomly divided into three groups on day 115 of pregnancy: without treatment (*N* = 50), with 75 μg *D*-cloprostenol sodium (*N* = 137), and with 200 μg *DL*-cloprostenol sodium (*N* = 133). After delivery, the sows treated with *D*-cloprostenol sodium and *DL*-cloprostenol sodium were randomly allocated into three subgroups, respectively: (i) no additional treatment after farrowing; (ii) administration of cloprostenol sodium at 3 h and 5 days after farrowing; and (iii) administration of cloprostenol sodium at 3 h, 5 days, and 10 days after farrowing. Cloprostenol sodium effectively induced sows to synchronize parturition approximately 23 h after administration and increased the daytime delivery rates (*p* < 0.05). Compared with *DL*-cloprostenol sodium, *D*-cloprostenol sodium shortened the farrowing duration and birth interval of sows for inducing farrowing (*p* < 0.05). Moreover, we observed that a single administration of both *D*-cloprostenol sodium and *DL*-cloprostenol sodium a day before delivery significantly reduced the rates of stillborn piglets type II in MS (*p* < 0.05). Compared to no treatment and single treatment with cloprostenol sodium, quartic treatments with cloprostenol sodium significantly increased the daily feed intake of MS, litter weight after weaning, and average daily gain of piglets (*p* < 0.05). Cloprostenol sodium improved the 21-day milk yield, with *D*-cloprostenol sodium showing the best effect, which increased lactation ability by 30.30% (176.72 kg vs. 135.63 kg) (*p* < 0.05). *DL*-cloprostenol sodium followed closely, increasing lactation ability by approximately 25.00% (169.71 kg vs. 135.63 kg) (*p* < 0.05). During lactation, sows administered with *D*-cloprostenol sodium observed increased serum prolactin levels. Compared to untreated sows, the sows administered with *D*-cloprostenol sodium and multiple *DL*-cloprostenol sodium visibly shortened the weaning-to-estrus interval (WEI) and weaning-to-service interval (WSI) (*p* < 0.05). Furthermore, quartic injections of *D*-cloprostenol sodium resulted in an 18 percentage point increase in the pregnancy rate of breeding sows compared to controls (82.61% vs. 64.58%) (*p* > 0.05). In summary, cloprostenol sodium could enhance the reproductive performance of MS, particularly in terms of lactation performance. Additionally, the effect of quartic injections of *D*-cloprostenol sodium was the most pronounced.

## 1 Introduction

The lactation ability of sows is the most crucial factor affecting piglet growth rate and the number of piglets weaned per sow per year (PSY). The analysis of the milk yield from a pig farm in Denmark revealed that approximately 23.5% of colostrum (the milk within 24 h postpartum) yield were less than 5 kg, while only 30% of the sow’s colostrum yield exceeded 7 kg (1). The colostrum yield dose was not equal to the colostrum intake of sucking piglets, and failure to obtain sufficient colostrum prior to weaning is one of the most important causes of piglet mortality. Hassan et al. found that 23.5% of piglets had a colostrum intake below 200 g, while 36% had a colostrum intake below 250 g (2). When the colostrum intake of piglets is less than 200 g, the pre-weaning mortality rates can reach as high as 43.3%; however, pre-weaning mortality can be reduced to 7.1% when the colostrum intake of piglets was more than 200 g (3). Induced parturition may affect colostrum yield in sows. The administration of oxytocin significantly reduces both the colostrum yield in sows and the colostrum intake by piglets, while PGF_2α_ analogs compensate for the deficiency of oxytocins (4). The timing of induced parturition also affects the composition of colostrum. Early initiation of delivery using cloprostenol sodium reduces the levels of IgG and total protein in colostrum (5).

The milk yield of the sows fails to satisfy the energy and protein requirements of piglets (9–10 pigs/litter) beyond day 8 of lactation, resulting in a gradual widening of this gap throughout lactation; as a result, milk production only meets approximately 50% of the needs of 21-day-old piglets for maximum growth (6). Stimulating mammary gland development represents the most practical approach to enhance milk yield in sows. Rapid breast development consists of three stages. The initial stage of rapid development occurs from 90 days of age until puberty and has received limited research attention to date. The second stage occurs during the one-third period after gestation and is the mature stage. The third stage occurs within the first 2 weeks of lactation, with limited studies available on this phase (7). Lactation deficiency is caused by both hereditary and acquired endocrine disorders (8). It has been reported that continuous injection of recombinant pig prolactin (PRL) for 28 days in prepubertal gilts weighing 75 kg can increase the content of parenchymal tissue, dry matter, total protein, and total DNA by upregulating the mRNA levels of PRL receptor (PRLR), STAT5A, and STAT5B in mammary tissues; thus, this treatment effectively promotes mammary gland development in gilts (9). An increasing number of studies aim to regulate the lactation performance of sows by exogenous hormones. The gilts were injected with 5 mg/day of porcine growth hormone on day 89 of pregnancy, and their mammary glands were collected for analysis on day 110 of pregnancy. The results showed that the treatment group exhibited a significant increase in the mass of mammary gland parenchyma (1,922.2 g vs. 1,576.1 g), and the mammary gland parenchyma of the treatment group contained more protein, glucose, DNA, and RNA (10). Furthermore, exogenous hormones such as estradiol and PGF_2α_ have been demonstrated to induce lactation in 81.3% of non-pregnant sows within 38–59 days after mating (11).

Maternal factors potentially affecting lactation include parity, preterm birth, dystocia, and residual placental fragments (8). PGF_2α_ serves as the main luteolytic factor (12), which can dissolve the corpus luteum and contract uterine smooth muscle. Consequently, PGF_2α_ is frequently used for estrus and farrowing synchronization in livestock. Cloprostenol sodium is one of the most important synthetic analogs of PGF_2α_, which is mainly eliminated by the lungs and kidneys with a half-life of up to 3 h (13, 14). Cloprostenol sodium (*DL*-cloprostenol sodium) exists as two optical isomers, which can be divided into *D*-isomer and *L*-isomer according to the relative configuration of the chiral carbon atoms. They are named *D*-cloprostenol sodium and *L*-cloprostenol sodium, respectively. The proportions of the two enantiomers in cloprostenol sodium are equal (15). *DL*-cloprostenol sodium has been widely used in livestock production for many years, while *D*-cloprostenol sodium has not been extensively used. Experimental evidence showed that *D*-cloprostenol sodium exhibited approximately three to four times greater efficacy in initiating luteal dissolution compared to *DL*-cloprostenol sodium, indicating that *L*-cloprostenol sodium may have no effect on luteal lysis or even demonstrate a reverse effect (15).

In light of the above findings, PGF_2α_ analogs, as uterine contractile agents, have been shown to accelerate placental discharge, reduce the incidence of uterine inflammation, and also enhance delivery performance and colostrum composition in sows. Although *DL*-cloprostenol sodium and *D*-cloprostenol sodium belong to PGF_2α_ analogs, their effects on lactating performance in sows have not been reported. The first 2 weeks of lactation are a crucial stage for promoting breast development and lactation. Meanwhile, as piglets grow, sows’ milk in the middle and late stages of lactation may not adequately meet their growth and development needs. Consequently, we designed this experiment to investigate the effects of induced parturition before farrowing and injection with either 75 μg/time *D*-cloprostenol sodium (at the full-recommended dosage) or 200 μg/time *DL*-cloprostenol sodium (at the full-recommended dosage) at 3 h, 5 days, and 10 days postpartum on reproductive performance, milk quality, estrous cycle, serum lactation-related hormones in multiparous sows (MS), and the lactation growth performance of offspring piglets during lactation. These results may provide valuable technical guidance for the utilization of PGF_2α_ analogs, especially *D*-cloprostenol sodium, in sow lactation management.

## 2 Materials and methods

### 2.1 Experimental design

This study was conducted in a swine breeding herd consisting of 2,000 sows located in northern Guangdong between June and September 2022. The average daily minimum and maximum temperatures during the experimental period ranged from 28.0°C to 35.0°C. The 320 Yorkshire × Landrace MS randomly selected for this study had the same genetic background, a parity of 2–3, and similar body weights (approximately 185–250 kg), back fat thickness (approximately 14–23 mm), litter size (6–18 alive piglets), and other reproductive performance. The experimental MS were obtained from Guangdong Guanghui Huifeng Farm (Shaoguan City, Guangdong Province, China). They were reared in a conventional semi-open housing system. The experimental sows were transferred to the farrowing house 5–7 days prior to the estimated farrowing date. They were individually housed in crates until the weaning period, which occurred 21 ± 1 days after farrowing.

**Figure 1:**
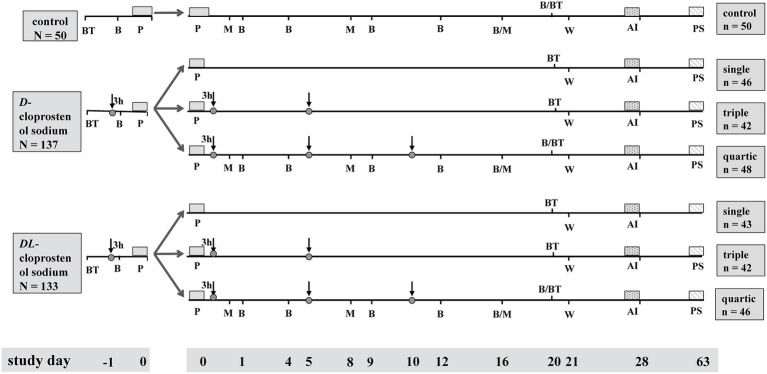
Diagram of activities and protocol for the synchronization of parturition and timed treatment, sampling, examining, weaning, and artificial insemination. Control, multiparous sows not supplemented with cloprostenol sodium; *D*-cloprostenol sodium, the sows treated with 75 μg *D*-cloprostenol sodium; *DL*-cloprostenol sodium, the sows injected with 200 μg *DL*-cloprostenol sodium. The BT in diagram represents the evaluation of sows’ back fat thickness at 110 days of gestation and 20 days postpartum. The ↓ in the diagram indicates the time of administration of *D*-cloprostenol sodium or *DL*-cloprostenol sodium. The B and M in drawing show the time of blood sampling and milk sampling, respectively. The P, W, AI, and PS in figure depict the time of parturition, weaning, artificial insemination, and pregnancy testing, respectively. The single, triple, and quartic indicate the frequency of *D*-cloprostenol sodium or *DL*-cloprostenol sodium administration to multiparous sows.

The experimental sows were classified into three groups based on the dose of *D*-cloprostenol sodium or *DL*-cloprostenol sodium administered via an intramuscular injection in the neck between 11:30 a.m. and 12:00 a.m. 1 day prior to the expected delivery date of MS: Some sows underwent natural farrowing (*N* = 50), whereas the remaining received a single administration of 75 μg (1 mL) *D*-cloprostenol sodium (75 μg/mL, 1 mL/sow, Ningbo Second Hormone Factory, Zhejiang Province, China; *N* = 137) and 200 μg (2 mL) *DL-*cloprostenol sodium (100 μg/mL, 2 mL/sow, Ningbo Second Hormone Factory, Zhejiang Province, China; *N* = 133) (Figure 1). Sows that had begun to deliver before 11:30 a.m. on day 115 of pregnancy were excluded from the experiment. The original litter weight and litter sizes of the piglets were assessed immediately after parturition. The sow injected with *D*-cloprostenol sodium (*n* = 1) and *DL*-cloprostenol sodium (*n* = 1) died due to dystocia during parturition. The occurrence of postpartum paralysis was observed in one sow after the injection of *DL*-cloprostenol sodium, while no clinical signs were reported in the others.

To evaluate the impact of cloprostenol sodium on lactation in MS, piglets were appropriately cross-fostered within the group within 3 h after delivery. However, piglets that were too small (< 0.8 kg) and/or too weak (unable to stand or crawl) were swapped with non-trial piglets (0.8–1.2 kg), which ultimately ensured that the number of piglets per litter averaged approximately 10, with a balanced gender ratio that was essentially identical. For the milk performance study, the remaining sows that did not receive any oxytocin during farrowing with *D-*cloprostenol sodium (*N* = 136) were randomly allocated into three subgroups: (i) the sows were not additionally treated postpartum (*n* = 46); (ii) the sows were administered double doses of 75 μg/time *D*-cloprostenol sodium at 3 h and 5 days after the end of farrowing, respectively (*n* = 42); and (iii) the sows were administered triple doses of 75 μg/time *D*-cloprostenol sodium at 3 h, 5 days, and 10 days after the end of farrowing, respectively (*n* = 48) (Figure 1). In all subgroups, the number of live piglets per litter and the weight of each litter were uniformly consistent.

The sows injected with *DL*-cloprostenol sodium received the same treatment. The remaining sows (*N* = 131) were randomly allocated into three subgroups: (i) the sows were not additionally treated after farrowing (*n* = 43); (ii) the sows were administered 200 μg/time *DL*-cloprostenol sodium at 3 h and 5 days after the end of farrowing, respectively (*n* = 42), and (iii) the sows were administered 200 μg/time *DL*-cloprostenol sodium at 3 h, 5 days, and 10 days after the end of farrowing, respectively (*n* = 46) (Figure 1). In all subgroups, the number of live piglets per litter and the litter weight were also consistent, respectively. During the pre-weaning growth period, piglets were unable to undergo cross-fostering among different subgroups.

### 2.2 Parturition process monitoring and postpartum management

Sows gave birth in calm environments. Two research teams, consisting of a total of six individuals, carefully supervised the parturition process for 24 h and ensured that there was at least one recording personnel in each farrowing house to record the timing of each piglet’s birth and the expulsion of each placenta in the sow. Parturition duration was defined as the period from the first to the last piglet delivery, and placental expulsion duration was defined as the period from the first to the last placental expulsion. Birth assistance was provided at an interval of >60 min after the parturition of the previous piglet or when the sows did not give birth to the first piglet for >4 h after breaking the amniotic fluid. Birth assistance included applying pressure to the sows’ belly and manually extracting the fetus or placenta. The method of pressing the belly was as follows: First, the sow’s udders were gently massaged using sterilized bare feet, inducing a constricted appearance of the sow’s body and resulting in an arched belly. Subsequently, pressure was slowly applied from the front of the arch (close to heart) to the back (close to tail), which stimulated sustained power in the sow and promoted rapid piglet production. The administration of oxytocin should be avoided during the process of inducing farrowing. The total number of piglets born, the number of piglets born alive, the number of piglets born weak, and the number of stillborn piglets were also recorded at farrowing. Stillbirths can be classified into two types based on the time of death (16). Stillborn piglet type I includes deaths that occurs before the sow delivers and is most commonly attributed to infectious causes. The fetal death exhibits dull, pale skin without luster or a rotten body. Stillborn piglet type II is often associated with dystocia and intrauterine and birth canal asphyxia during parturition. The fetal death is characterized by a fresh, ruddy skin and an extended tongue in a state of asphyxia, with the mouth and nose obstructed by mucus.

Daytime was defined as the period of 0800–2,200 h based on the day-shift working time of the midwifery technical personnel, and the nighttime was defined as the period of 2,200–0800 h. If the sow delivered exactly at 0800 or 2,200 h, it would be divided according to the period when most piglets in the litter were born. At the end of the farrowing process, all MS were injected with a non-steroidal anti-inflammatory drug (tolfenamic acid, 2 mg/kg, Sivea (Qingdao) Bio pharmaceutical Co., Ltd., Shandong Province, China), an antibiotics (ceftiofur, 10 mg/kg, Zoetis Suzhou Biopharmaceutical Co., Ltd., Jiangsu Province, China), and a multivitamin (vitamin A, D and E; 10 mL/sow; Hebei Yuanzheng Pharmaceutical Co., Ltd., Hebei Province, China) through the neck muscles on days 1–3 postpartum. Piglets received the same care and supervision according to pig farm regulations, which included tail docking, tooth clipping on the first day of life, and intramuscular administration of iron supplement (iron dextran, 1 mL/piglet, Sivea (Qingdao) Bio pharmaceutical Co., Ltd., Shandong Province, China) on the third day of life. The sows were provided with the same feed and drinking water according to the management of the pig farms.

### 2.3 Collection and determination of serum hormones and milk components

In the quartic group, which includes the quartic *D*-cloprostenol sodium and *DL*-cloprostenol sodium groups, as well as the control group, three sows were randomly selected from each group for blood sample collection via the anterior jugular vein. Blood was collected from adult sows using the standing fixed method. The breeder lifted the upper jaw of the sow with a fixed rope to expose both sides of the prethoracic fossa. Another breeder put a needle in the direction of the low and vertical concave bottom of the right prethoracic fossa, took the required amount of blood, pressed the needle site with a cotton ball to stop bleeding, and relieved the sow. During the course of the study, a total of seven blood collections were performed on the selected sows, with 3 mL of blood samples being obtained in each instance. The day of delivery was designated as day 0. The initial blood samples were conducted at 1,500 h on day 115 of the sows’ gestation (day −1), 3 h post-administration of the first dose. Other blood samples were collected at 0900 h on days 1, 4, 9, 12, 16, and 20 after farrowing (Figure 1). After collection, all blood samples were incubated at room temperature (30 ± 2°C) for 10 min, followed by centrifugation at 4,000 r/min for 20 min. The serum was immediately sub-packed and transferred to −20°C for preservation. Serum samples were sent on dry ice to Beijing North Institute of Biotechnology Co., Ltd. to detect the concentrations of estradiol-17β (E_2_), PRL, and cortisol (COR) by radioimmunoassay and progesterone (P_4_) using enzyme-linked immunosorbent assay.

Three sows, excluding the sows selected for blood collection, were randomly selected for milk collection from the quartic *D*-cloprostenol sodium group, quartic *DL*-cloprostenol sodium group, and control groups (Figure 1). The milk samples were collected three times, with each collection consisting of 2 mL. The colostrum was collected within a period of 12 h after farrowing, and mature milk was obtained by administering an injection of 10 IU (1 mL) oxytocin (10 IU/1 mL, 1 mL/sow, Ningbo Second Hormone Factory, Zhejiang Province, China) in the neck on 8 and 16 days post-farrowing. Milk samples were dispatched to Shanghai Enzyme Union Biotechnology Co., Ltd. under dry ice conditions for the purpose of quantifying fat, protein, and lactose concentrations through infrared spectroscopy.

### 2.4 Back fat and daily feed intake determination in MS

The back fat thickness of all MS was evaluated upon entering the farrowing house and on 20 days postpartum, utilizing an Abdominal Convex Probe and a Veterinary B-mode ultrasonic diagnostic instrument (WED-3000 V, Shenzhen Well. D Medical Electronics Co., Ltd., Guangdong Province, China). Each pig is measured three times simultaneously, and the average value was recorded. An ultrasound probe was placed approximately 6.5 cm from the dorsal midline on the last rib curve to measure the back fat thickness. The lactational back fat loss was calculated as the difference between back fat thickness when entering the farrowing house and on day 20 of lactation (Figure 1).

The daily feed intake of the MS was recorded from delivery until day 21 of lactation. The farrowing house is equipped with automatic feeders for each crate, ensuring precise quantification of the amount of feed for each individual feeding. Postpartum sows were fed twice a day at 0800 h and 1,700 h. On the day of parturition (day 0), an initial feeding amount of 0.5 kg was provided, followed by a daily increment of 1 kg until day 5. Subsequently, the feeder scale was adjusted based on each sow’s previous intake to optimize and maximize *ad libitum* feeding until weaning. It is worth noting that the remaining feed in the tank needs to be weighed and recorded before each feeding. These feed refusals should be summarized at weaning. Subsequently, a statistical analysis was conducted to determine the daily feed intake of each sow from parturition until weaning.

### 2.5 Statistics of litter weaned weight and estimation of milk power

We collected the weights of newborn litter and weaning litter on day 21 of lactation. The average daily weaning gain (ADWG, g/d) of the piglets from birth to weaning was calculated, as shown in Equation (1). The lactation ability refers to the algorithm of Lawlor et al. (17) as shown in Equation (2).


(1) $$\mathrm{ADWG}\;\left(g/d\right)=\left[\begin{array}{l} \mathrm{average}\;\mathrm{body}\;\mathrm{weight}\;\mathrm{at}\;\mathrm{weaning}\;\left(g\right)\\ -\mathrm{average}\;\mathrm{body}\;\mathrm{weight}\;\mathrm{at}\;\mathrm{birth}\;\left(g\right) \end{array}\right]/21\;\mathrm{days}$$

(2) $$\begin{array}{c} 21-\mathrm{day}\;\mathrm{milk}\;\mathrm{yield}\;\left(\mathrm{kg}\right)=\mathrm{piglets}\;\mathrm{of}\;\mathrm{ADWG}\;\left(g/d\right)\times 21\;\mathrm{days}\\ \;\times \mathrm{number}\;\mathrm{of}\;\mathrm{weaned}\;\mathrm{piglets}\\ \;\times 4/1000 \end{array}$$

### 2.6 Statistics of estrus after 7 days of weaning

The transfer of all MS from the parturition housing to the mating housing was completed on day 21 of lactation. According to regulations for the pig farm, boars were migrated twice daily to the mating housing for stimulation after weaning to induce sows’ estrus. Data regarding the timing of estrus and artificial insemination (AI), the number of estrus and mating events within 7 days of weaning, and the number of pregnancies from 28 to 35 days after mating in weaned MS were recorded (Figure 1).

### 2.7 Statistical analyses

Huifeng Farm procedure data were organized using Excel 2013 software (Microsoft, Redmond, Washington, USA). Statistical analyses were performed using the GLM procedure of IBM SPSS Statistics 20 software (IBM, Armonk, NY, USA) and a two-way ANOVA of GraphPad Prism software (Santiago, CA, USA). For the experimental investigation of synchronized delivery, the GLM procedure included the different treatments (blank drug, *D*-cloprostenol sodium, and *DL*-cloprostenol sodium) were taken as fixed effects, and back fat thickness at 110 days of gestation served as a covariate. Farrowing duration, birth interval, placenta expulsion duration, gestation length, administration-to-delivery intervals, litter size, and other indicators were treated as response variables;

Similarly, for the purpose of the lactation experiment, GLM included different treatments, the number of treatments [zero, single, triple and quartic injection(s)], and the interaction effects between them, all of which were taken as fixed effects. The pre-delivery back fat thickness was utilized as a covariable to examine the response variables, including daily feed intake, number of weaned piglets, total litter weight at weaning, average piglet weight at weaning, piglets of ADWG, 21-day milk yield, weaning back fat thickness, back fat loss, weaning-to-estrus interval (WEI), and weaning-to-service interval (WSI).

In addition, employing back fat thickness at 110 days of gestation as a covariate, we analyzed binary dependent variables such as estrus rates, conception rates, daytime delivery rates, rates of piglets with birth interval > 30 min, farrowing assistance rates, Stillborn Type I and Type II rates, and weak piglets rates using logistic regression model. GraphPad Prism software was employed to generate all line charts and bar charts, and the discrepancies were examined through a two-way ANOVA. The results were expressed as mean ± SD or percentage. Statistical significance was set at a *p* of <0.05.

## 3 Results

### 3.1 Delivery process

Delivery data were collected from 320 sows during the experiment (Table 1). In our experiment, oxytocin was not used to assist with the delivery. The administration of cloprostenol sodium synchronized the farrowing duration of the sows (Table 1). Compared with the control sows (from 1,200 h on day 115 of gestation until delivery), the sows induced by *D*-cloprostenol sodium and *DL*-cloprostenol sodium exhibited more concentrated delivery approximately 23 h after administration, resulting in significantly reduced administration-to-delivery intervals of sows (*p* < 0.05). Administration started on day 115 of gestation at 1,130–1,200 h, followed by concentrated delivery from 1,000 to 1,100 h the next day. Additionally, in comparison with the control sows (42.00%), the sows induced *D*-cloprostenol sodium (92.70%) and *DL*-cloprostenol sodium (87.22%) exhibited significantly higher daytime delivery rates (*p* < 0.05). The treatment sows exhibited significantly shortened farrowing duration, birth interval, and placenta expulsion duration in comparison with the control sows (*p* < 0.05). The farrowing duration (182.95 min vs. 217.33 min, *p* < 0.05) and birth interval (17.89 min vs. 21.61 min, *p* < 0.05) were shorter when using *D*-cloprostenol sodium in comparison with *DL*-cloprostenol sodium. However, placenta expulsion duration induced by *D*-cloprostenol sodium did not show any significant differences compared to *DL*-cloprostenol sodium (*p* < 0.05).

**Table 1:** Effect of different cloprostenol sodium on the process of synchronized delivery in parturient sows (Means ± SD & Percentage).

Groups ^1^	Control	*D*-cloprostenol sodium	*DL*-cloprostenol sodium	*p*-value
Number of sows	50	137	133	/
Gestation length, d	117.02 ± 1.02^a^	115.91 ± 0.38^b^	115.95 ± 0.46^b^	< 0.001
Administration-to-delivery intervals, h^2^	48.94 ± 26.68^a^	22.93 ± 7.58^b^	23.48 ± 8.52^b^	< 0.001
Farrowing duration, min^3^	305.70 ± 171.88^a^	182.95 ± 73.16^c^	217.33 ± 87.94^b^	< 0.001
Birth interval, min	31.08 ± 19.61^a^	17.89 ± 6.49^c^	21.61 ± 7.91^b^	< 0.001
Placenta expulsion duration, min^4^	254.56 ± 87.47^a^	180.72 ± 62.08^b^	194.51 ± 78.02^b^	< 0.001
Piglets with birth interval > 30 min, %	42.00^a^	8.00^b^	14.29^b^	< 0.001
Farrowing assistance, %	10.00	8.03	15.03	0.342
Sow mortality at dystocia, %	0.00	0.73	0.75	0.454
Daytime delivery, %^5^	42.00^b^	92.70^a^	87.22^a^	< 0.001

^1^Multiparous sows underwent natural farrowing (control group), and sows treated with a single dose of 75 μg *D*-cloprostenol sodium or 200 μg *DL*-cloprostenol sodium were induced to undergo parturition. ^2^No additional medication was given in control group, and the time from 1,200 h on day 115 of pregnancy to delivery was defined as the administration-to-delivery intervals. Other groups record according to the actual situation. ^3^The period from the first to the last piglet delivery. ^4^The period from the first to the last placental expulsion. ^5^Daytime was defined as 0800–2,200 h based on the day-shift working time of the midwifery technical personnel, and the nighttime was defined as 2,200–0800 h. If the sow was delivered at exactly 0800 or 2,200 h, it was divided according to the period when most piglets in the litter were born. ^a,b,c^Different superscripts within rows differ significantly (*p* < 0.05).

The incidence of piglets with birth intervals >30 min in sows treated with *D*-cloprostenol sodium (8.00%) and *DL*-cloprostenol sodium (14.29%) was significantly lower than that in the control sows (42.00%) (*p* < 0.05). Birth assistance was observed for each group (*p* = 0.342): control group (10.00%), *D*-cloprostenol sodium group (8.03%), and *DL*-cloprostenol sodium group (15.03%). In contrast, the incidence of birth assistance in sows appeared to be reduced with *D*-cloprostenol sodium; however, no statistically significant difference was observed compared to the treatment of *DL*-cloprostenol sodium (*p* > 0.05). One dystocia that resulted in non-survival occurred in each sow’s induced parturition, one with *D*-cloprostenol sodium, and one with *DL*-cloprostenol sodium. No deaths occurred in the control sows. However, no significant differences were observed among the groups (*p* = 0.454).

### 3.2 Newborn piglets’ characteristics

The litter sizes of sows naturally farrowing compared with those treated with cloprostenol sodium approximately 23 h before farrowing (referred to as 24 h before farrowing for convenience) are shown in Table 2. Compared to the control group (5.76%), the incidence of Stillborn Type II was lower in sows treated with *D*-cloprostenol sodium (1.74%) and *DL*-cloprostenol sodium (3.13%) (*p* < 0.05). Additionally, the rate of Stillborn Type I in the *D*-cloprostenol sodium group was less than that in the *DL*-cloprostenol sodium group (*p* < 0.05). There were no significant differences observed among all groups regarding rates of born weak piglets, Stillborn Type I (*p* > 0.05).

**Table 2:** Effect of synchronized delivery induced by different cloprostenol sodium on litter performance of multiparous sows (Means ± SD & percentage).

Groups^1^	Control	*D*-cloprostenol sodium	*DL*-cloprostenol sodium	*p*-value
Number of sows	50	136	132	/
Number of piglets^2^	573	1,550	1,500	/
Total number of born piglets	11.46 ± 1.78	11.40 ± 2.20	11.36 ± 2.29	0.978
Number of born alive piglets	10.28 ± 1.28	10.44 ± 1.26	10.29 ± 1.18	0.510
Number of born strong piglets^3^	10.00 ± 1.26	10.13 ± 1.32	9.77 ± 1.53	0.150
Born weak piglets, %^4^	3.32	2.52	2.87	0.594
Stillborn piglet type I, %^5^	2.97	2.52	3.07	0.638
Stillborn piglet type II, %^6^	5.76^a^	1.74^c^	3.13^b^	< 0.001
Total litter weight at birth, kg	14.93 ± 2.63	15.35 ± 2.65	15.05 ± 2.64	0.492
Average piglet weight at birth, kg	1.46 ± 0.23	1.48 ± 0.23	1.47 ± 0.23	0.875

^1^Multiparous sows underwent natural farrowing (control group), and sows treated with a single dose of 75 μg *D*-cloprostenol sodium or 200 μg *DL*-cloprostenol sodium were induced to undergo parturition. ^2^Total number of piglets delivered by sows in each group. ^3^Piglets that are alive, weigh equal to or greater than 800 g, and suckle colostrum normally. ^4^Piglets that weigh less than 800 g or do not suckle colostrum. ^5^Fetal death occurs before the sow delivers and includes mummified piglets. ^6^Fetal death, occurring during a sow’s parturition, is attributed to asphyxia within the birth canal and intrauterine. ^a,b,c^Different superscripts within rows differ significantly (*p* < 0.05).

After farrowing, the MS treated with *D*-cloprostenol sodium and *DL*-cloprostenol sodium were randomly allocated into three subgroups (Figure 1). Remarkably, one sow exhibited postpartum paralysis symptoms in the *DL*-cloprostenol sodium group; thus, this sow would be excluded from subsequent trials. On average, there were no differences observed in back fat thickness on day 110 of gestation, adjusted litter size, and litter weight (Tables 3, 4).

**Table 3:** Effects of different injections of cloprostenol sodium on back fat and daily feed intake in sows during lactation (Means ± SD).

Variables	Control^1^	*D*-cloprostenol sodium^2^			*DL*-cloprostenol sodium^3^			*p*-values^4^		
		Single	Triple	Quartic	Single	Triple	Quartic	Drug	Time	Int
Number of sows^5^	50	46	42	48	43	42	46	/	/	/
Back fat thickness on day 110 of gestation, mm^6^	17.07 ± 4.53	16.70 ± 4.86	17.48 ± 6.97	17.31 ± 6.11	16.77 ± 5.30	18.10 ± 6.56	17.01 ± 4.81	/	/	/
Number of weaned sows	48	46	41	46	42	41	45	/	/	/
Back fat thickness on day 20 of lactation, mm	15.19 ± 4.34	14.68 ± 4.22	15.79 ± 5.10	15.42 ± 4.91	14.42 ± 3.99	16.07 ± 5.41	14.94 ± 4.48	0.594	0.768	0.986
Back fat loss, mm	2.03 ± 4.13	2.01 ± 2.36	1.85 ± 3.27	1.88 ± 2.70	2.07 ± 3.34	2.12 ± 2.83	2.18 ± 1.74	0.594	0.768	0.986
The daily feed intake, kg	5.07 ± 0.69^b^	5.25 ± 0.65^b^	5.71 ± 0.57^a^	5.83 ± 0.74^a^	5.16 ± 0.66^b^	5.61 ± 0.56^a^	5.64 ± 0.76^a^	0.121	< 0.001	0.630

^1,2,3^Multiparous sows without any additional treatment (control). Sows were administered a single dose of 75 μg/time *D*-cloprostenol sodium or 200 μg/time *DL*-cloprostenol sodium, which was induced 24 h before delivery (Single); sows were administered a triple dose of cloprostenol sodium, 24 h before delivery, and again 3 h and 5 days after delivery (Triple); sows were administered four doses of cloprostenol sodium, 24 h before delivery, and again 3 h, 5 days, and 10 days after delivery (Quartic). ^4^*p*-values include main effects of drugs (control or cloprostenol sodium) and times of treatments (Time) and the interaction between drug and times (Int). ^5,6^After parturition, sows treated with *D*-cloprostenol sodium and *DL*-cloprostenol sodium were randomly divided into three groups based on different postpartum treatment methods. The back fat thickness of sows in each subgroup was recorded before parturition. ^a,b^Different superscripts within rows differ significantly (*p* < 0.05).

**Table 4:** Effects of different injections of cloprostenol sodium on litter weight of weaned piglets and milk yield of sows during lactation (Means ± SD).

Variables	Control ^1^	*D*-cloprostenol sodium^2^			*DL*-cloprostenol sodium^3^			*p*-values^4^		
		Single	Triple	Quartic	Single	Triple	Quartic	Drug	Time	Int
Number of sows^5^	50	46	42	48	43	42	46	/	/	/
Litter size^6^	10.34 ± 1.19	10.43 ± 1.36	10.44 ± 1.27	10.48 ± 1.89	10.40 ± 1.43	10.34 ± 1.01	10.21 ± 1.04	/	/	/
Litter weight, kg^7^	15.04 ± 2.51	15.31 ± 2.72	15.42 ± 2.74	15.38 ± 2.56	15.08 ± 2.46	15.45 ± 3.07	14.68 ± 2.37	/	/	/
Average piglet weight, kg^8^	1.46 ± 0.22	1.47 ± 0.23	1.48 ± 0.22	1.47 ± 0.23	1.47 ± 0.25	1.51 ± 0.28	1.45 ± 0.24	/	/	/
Number of weaned sows	48	46	41	46	42	41	45	/	/	/
Number of weaned piglets	9.00 ± 1.22^b^	9.35 ± 1.04^ab^	9.59 ± 1.00^a^	9.80 ± 0.86^a^	9.36 ± 1.19^ab^	9.37 ± 0.86^ab^	9.49 ± 0.97^a^	0.227	< 0.001	0.643
Total litter weight at weaning, kg	47.07 ± 8.47^d^	51.55 ± 7.66^bc^	55.23 ± 11.04^ab^	58.83 ± 7.46^a^	50.18 ± 8.03^cd^	55.00 ± 8.40^ab^	55.83 ± 9.10^a^	0.192	< 0.001	0.663
Average piglet weight at weaning, kg	5.26 ± 0.87^c^	5.53 ± 0.67^bc^	5.75 ± 0.90^ab^	6.00 ± 0.57^a^	5.37 ± 0.56^c^	5.88 ± 0.76^a^	5.90 ± 0.89^a^	0.678	< 0.001	0.668
ADWG of piglets, g^9^	180.43 ± 35.72^c^	194.80 ± 31.03^bc^	203.24 ± 41.16^ab^	214.50 ± 26.23^a^	186.46 ± 25.32^c^	207.29 ± 31.91^ab^	213.88 ± 40.84^a^	0.767	< 0.001	0.665
21-day milk yield, kg^10^	135.63 ± 28.58^d^	152.67 ± 28.54^bc^	164.02 ± 39.84^ab^	176.72 ± 26.69^a^	146.24 ± 25.78^cd^	163.02 ± 29.01^ab^	169.71 ± 32.89^a^	0.264	< 0.001	0.818

^1,2,3^Multiparous sows without any additional treatment (control); sows were administered a single dose of 75 μg/time *D*-cloprostenol sodium or 200 μg/time *DL*-cloprostenol sodium, which was induced 24 h before delivery (Single); sows were administered a triple dose of cloprostenol sodium, 24 h before delivery and again 3 h and 5 days after delivery (Triple); sows were administered four doses of cloprostenol sodium 24 h before delivery and again 3 h, 5 days, and 10 days after delivery (Quartic). ^4^*p*-values include main effects of drugs (control or cloprostenol sodium) and times of treatments (Time) and the interaction between drug and times (Int). ^5,6,7,8^After parturition, to evaluate the impact of cloprostenol sodium on lactation in MS, piglets underwent appropriately cross-fostered within the group within 3 h after delivery. However, piglets that were too small (< 0.8 kg) and/or too weak (unable to stand or crawl) could be swapped with non-trial piglets (0.8–1.2 kg). The number and weight of piglets suckled by sows in each subgroup were recorded. ^9^Per piglets from birth to weaning daily weight. ADWG, Average Daily Weaning Gain. ^10^The 21-day milk yield refers to the algorithm of Lawlor et al. (17). ^a,b,c,d^Different superscripts within rows differ significantly (*p* < 0.05).

### 3.3 Back fat change and daily feed intake of sows

The experiment excluded a total of eight sows due to agalactia, paralysis, or other diseases during lactation. One in each of the triple *D*-cloprostenol sodium, single *DL*-cloprostenol sodium, triple *DL*-cloprostenol sodium, and quartic *D*-cloprostenol sodium groups were excluded from the test of lactation ability. Additionally, two in each of the control and quartic *D*-cloprostenol sodium groups were excluded from the test data statistics. Daily feed intake and back fat loss of the sows in each group during lactation are shown in Table 3.

There were no significant differences in back fat thickness between sows on day 110 of gestation and day 20 of lactation (*p* > 0.05), and the observed back fat loss during lactation was not significant (*p* > 0.05). Moreover, the feed intake of sows exhibited an increase in proportion to the number of postpartum administration of cloprostenol sodium (Table 3). The mean daily feed intake of sows in the experimental group receiving triple and quartic doses of cloprostenol sodium was significantly higher compared to that of the sows not treated and administered with a single drug (*p* < 0.05). Under an identical number of treatments, no significant correlation was observed between daily food intake and drug type or dosage (*p* > 0.05).

### 3.4 Weaned piglets’ performances

The lactation capacity of MS was assessed based on body weight at weaning, and the corresponding results are shown in Table 4. The number of weaned piglets in the sows treated with quartic doses of cloprostenol sodium and triple doses of *D*-cloprostenol sodium was significantly higher than that in the control group (*p* < 0.05). Similarly, the total litter weight at weaning in the quartic treatments of *D*-cloprostenol sodium groups (58.53 kg) and *DL*-cloprostenol sodium groups (55.83 kg) was significantly higher than that in the single injection group, which was approximately 51 kg (*p* < 0.05). Additionally, triple doses of cloprostenol sodium resulted in heavier weights compared to the control group’s weight of 47.07 kg (*p* < 0.05). The administration of a single dose of *D*-cloprostenol sodium also resulted in a higher litter weight of piglets (*p* < 0.05).

The average piglet weight at weaning, piglets’s ADWG, and litter weight at weaning showed consistent outcomes of change with the increase of the number of cloprostenol sodium treatments. The average piglet weight at weaning and ADWG in the quartic cloprostenol sodium treatments were higher than those in the single drug treatment and not administration (*p* < 0.05). Utilization of triple cloprostenol sodium also resulted in a significant improvement in both average piglet weight and ADWG compared to the control group (*p* < 0.05). The same trend was observed for milk yield, which was calculated using Equation (2). All indices pertaining to weaned piglets are displayed in Table 4. Throughout the entire lactation period, the repeated quartic treatments exhibited the most favorable effect on the indicators of piglet weight and weight gain, followed by triple treatments and single treatment, respectively, and, finally, the control group without any additional treatment. In other words, the amount of weight gain in piglets during lactation was found to be solely associated with the frequency of administration of cloprostenol sodium (*p* < 0.05) but showed no significant correlation with its type (*p* > 0.05). Parturition induced with *D*-cloprostenol sodium, but not *DL*-cloprostenol sodium, had a positive effect on litter weight at weaning and 21-day milk yield (*p* < 0.05).

Conformably, compared to naturally lactating MS, the milk yield of sows that received repeated quartic doses of *D*-cloprostenol sodium was the highest, which showed a significant increase of 30.30% (176.72 kg vs. 135.63, *p* < 0.05). However, *DL*-cloprostenol sodium increased milk yield by 25.13% (169.71 kg vs. 135.63 kg, *p* < 0.05). Furthermore, triple treatments of cloprostenol sodium increased the milk yield by approximately 20% (*p* < 0.05). In addition, induced parturition with a single dosage of *D*-cloprostenol sodium also was found to significantly increase the yield milk by 12.56% (*p* < 0.05).

### 3.5 Content of milk components

**Figure 2:**
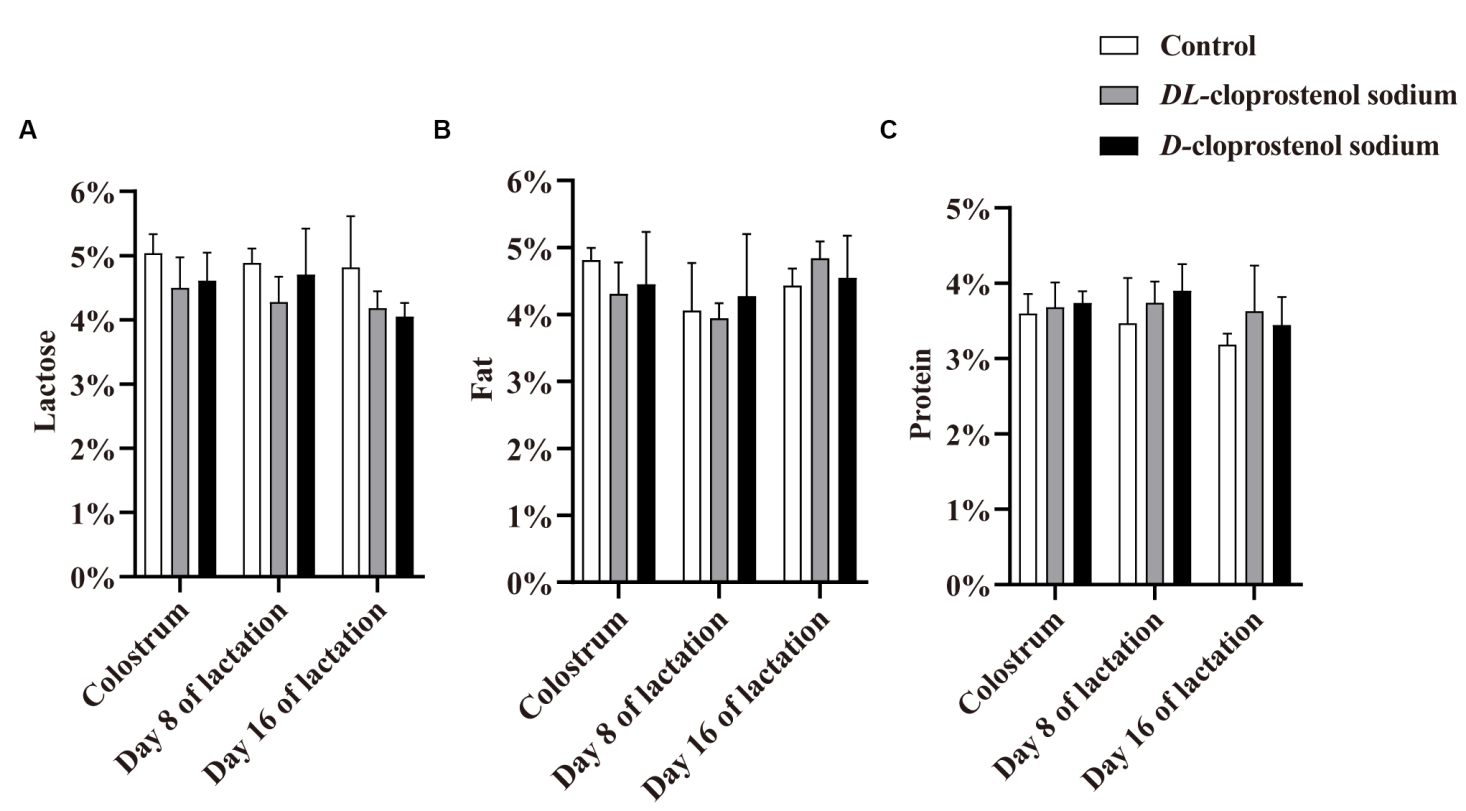
Concentration (%) of lactose **(A)**, fat **(B)**, and protein **(C)** in colostrum from multiparous sows within 12 h after parturition, as well as mature milk on the days 8 and 16 of lactation in both the control and treatment groups. Multiparous sows did not receive any additional treatment (Control, *n* = 3) and received 75 μg/time *D*-cloprostenol sodium (*n* = 3) and 200 μg/time *DL*-cloprostenol sodium (*n* = 3) quartic treatments, which were administered 24 h before delivery, 3 h and 5 days after delivery, and 3 h, 5 days, and 10 days after delivery, respectively.

The administration of cloprostenol sodium appeared to decrease the tendency of lactose content during the whole lactation period in sows (*p* > 0.05) (Figure 2). Similarly, the sows administered with double doses of cloprostenol sodium 24 h before farrowing and 3 h after farrowing resulted in a reduction in colostrum milk fat content; however, this difference did not reach statistical significance (*p* > 0.05). Nonetheless, the cumulative administration of treatment quartic injections resulted in an increase in mature milk fat content on day 16 of lactation in sows (*p* > 0.05) (Figure 2B). As shown in Figure 2C, triple and quartic injections of cloprostenol sodium exhibited a non-significant increase in milk protein content during lactation (*p* > 0.05). In summary, the cumulative treatment of cloprostenol sodium affected milk components in the colostrum and mature milk of sows in the early, middle, and late stages of lactation (*p* > 0.05).

### 3.6 Serum levels of lactation-related hormones

**Figure 3:**
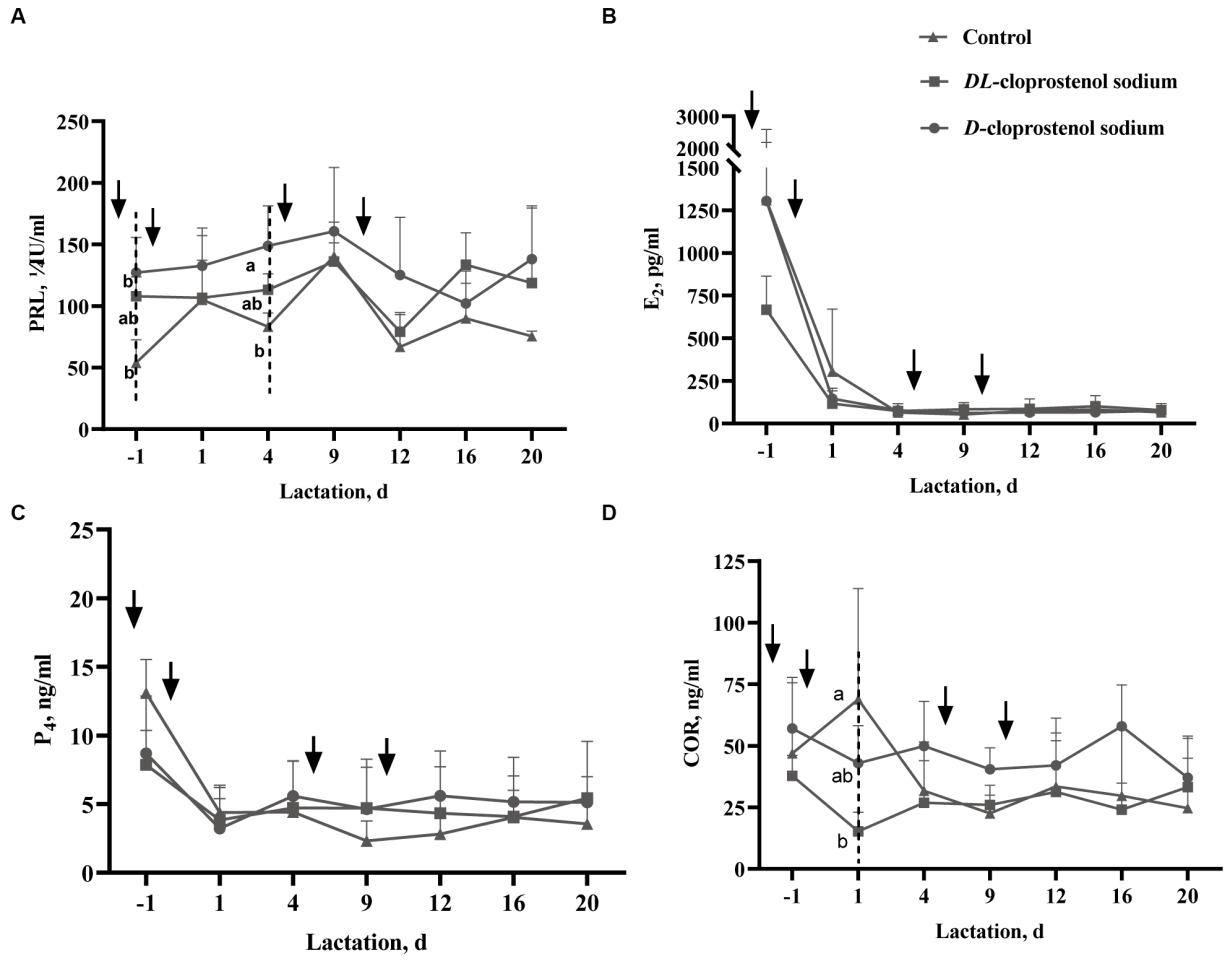
Effect of *D*-cloprostenol sodium or *DL*-cloprostenol sodium on serum prolactin (PRL) levels **(A)**, estradiol-17β (E_2_) levels **(B)**, progesterone (P_4_) levels **(C)**, and cortisol (COR) levels **(D)** in multiparous sows during lactation (Means ± SD). Multiparous sows without any additional treatment (*n* = 3) and those treated with 75 μg/time *D*-cloprostenol sodium (*n* = 3) and 200 μg/time *DL*-cloprostenol sodium (*n* = 3) were induced by quartic treatments 24 h before delivery, 3 h and 5 days after delivery, and 3 h, 5 days, and 10 days after delivery, respectively. The ↓ in the image indicates the time of administration of 75 μg *D*-cloprostenol sodium or 200 μg *DL*-cloprostenol sodium. The initial blood collection was conducted 3 h post the initial dosage, which merits mention. ^a,b^Different superscripts within the same time point of blood collection differ significantly (*p* < 0.05).

Figure 3A shows that the injection of *D*-cloprostenol sodium (*p* < 0.05) or *DL*-cloprostenol sodium (*p* > 0.05) on day 115 of gestation results in an increase in PRL levels in sows 3 h after the first administration. Additionally, the injection of *D*-cloprostenol sodium also led to a significant increase in PRL levels among lactating sows on day 4 (*p* < 0.05). Notably, both a single prenatal treatment and multiple postpartum treatments were found to elevate PRL levels during the lactation stage (Figure 3A). Receiving cloprostenol sodium treatment did not exert any significant impact on the serum estrogen levels in lactating sows (Figure 3B). Although a single dose cloprostenol sodium before parturition could reduce the levels of P_4_ in sows 3 h after treatment (*p* > 0.05), multiple doses of the drugs had no perceptible effect during lactations (Figure 3C). The sows treated with double cloprostenol sodium, especially *DL*-cloprostenol sodium, observed the lower COR levels on the first day of lactation. In addition, utilization of multiple doses of *D*-cloprostenol sodium exhibited a propensity to increase serum COR levels throughout lactation; however, the difference was not statistically significant (*p* > 0.05) (Figure 3D).

### 3.7 Reproductive performance of weaned sows

The effects of cloprostenol sodium treatment on the reproductive performance of next-parity sows are shown in Table 5. Although statistical significances were not observed among treatments (*p* > 0.05), the tendencies toward improvements of estrus rates and AI rate within 7 days post-weaning were noted with cloprostenol sodium intervention. It appeared that the weaning-to-estrus interval (WEI) was not only related to the optical activity (*p* < 0.05) but also to the number of treatments (*p* < 0.05). Compared to control sows, the sows treated with *D*-cloprostenol sodium exhibited the reduction in WEI (*p* < 0.05). Moreover, the sows administered *D*-cloprostenol sodium or *DL*-cloprostenol sodium showed the shorter WSI compared to the control group (*p* < 0.05). The conception rates of the next parity of weaned sows in quartic *D*-cloprostenol sodium (82.61%) were higher than those of the control group (64.58%) (*p >* 0.05). Similarly, other treatments also improved conception rates in sows, but there were no significant difference either (*p* < 0.05).

**Table 5:** Effects of different injection times of cloprostenol sodium on reproductive performance of weaned sows (Means ± SD & percentage).

Variables	Control^1^	*D*-cloprostenol sodium^2^			*DL*-cloprostenol sodium^3^			*p*-values^4^		
		Single	Triple	Quartic	Single	Triple	Quartic	Drug	Time	Int
Number of weaned sows	48	46	41	46	42	41	45	/	/	/
Estrus rate within 7 days after weaning, %	79.17	82.61	85.36	89.13	78.57	90.24	86.67	0.654	0.467	0.836
WEI, d^5^	4.16 ± 1.05^a^	3.55 ± 0.69^c^	3.63 ± 0.65^bc^	3.61 ± 0.67^bc^	3.97 ± 0.64^ab^	3.76 ± 0.68^bc^	3.85 ± 0.67^ab^	0.037	0.001	0.494
AI rate within 7 days after weaning, %^6^	68.75	73.91	78.05	84.78	71.43	78.05	82.22	0.487	0.304	0.949
WSI, d^7^	5.21 ± 0.89^a^	4.62 ± 0.70^b^	4.69 ± 0.64^b^	4.67 ± 0.58^b^	4.93 ± 0.74^bc^	4.72 ± 0.5^b^	4.78 ± 0.75^b^	0.189	< 0.001	0.636
Conception rate sows, %^8^	64.58	71.74	78.05	82.61	69.05	78.05	75.56	0.650	0.467	0.836

^1,2,3^Multiparous sows without any additional treatment (control); sows were administered a single dose of 75 μg/time *D*-cloprostenol sodium or 200 μg/time *DL*-cloprostenol sodium, which was induced 24 h before delivery (single); sows were administered a triple dose of cloprostenol sodium 24 h before delivery and again 3 h and 5 days after delivery (triple); sows were administered four doses of cloprostenol sodium 24 h before delivery and again 3 h, 5 days, and 10 days after delivery (quartic). ^4^*p*-values include main effects of drugs (control or cloprostenol sodium) and times of treatments (Time) and the interaction between drug and times (Int). ^5,6,7^WEI, weaning-to-estrus interval; AI, artificial insemination; WSI, weaning-to-service interval. ^8^The conception rate refers to the proportion of pregnant sows among weaned sows, rather than the proportion of mated sows. ^a,b,c^Different superscripts within rows differ significantly (*p* < 0.05).

## 4 Discussion

There is a divergence of opinions regarding the effects of PGF_2α_ and its analogs on the milk yield and milk composition in sows. Monteiro et al. (18) conducted a systematic analysis of eight articles to explain the effects of PGF_2α_ and its analogs on lactation in sows. Three of these articles reported the negative effects of PGF_2α_ drugs on lactation (5, 19, 20), three found that PGF_2α_ drugs had no significant effect on colostrum yield or piglet intake (4, 21, 22), one demonstrated that PGF_2α_ drugs could increase or decrease the content of certain cytokines to affect activity of colostrum (23), and one observed an increase in serum IgG concentration and weight gain in 3-day-old piglets (24). Previously, researchers have attempted to enhance the feed intake and milk yield of sows as well as the growth rate of piglets by employing various feed additives during the lactation period (25–27). This experiment aims to explore a novel pathway utilizing sex hormones to regulate the endocrine system and improve lactation performance during lactation. The effects of different injection times of PGF_2α_ analogs (75 μg *D*-cloprostenol sodium and 200 μg *DL*-cloprostenol sodium) on sows’ lactation ability are also discussed.

### 4.1 Sows of synchronous delivery

This study commenced with inducing parturition. The sows were administered with *D*-cloprostenol sodium or *DL*-cloprostenol sodium in the neck muscle to initiate synchronized delivery at 1,130–1,200 h on the day before the actual expected delivery of sows with a parity of 2–3. The findings showed that it could accelerate the delivery process, significantly shortening the time from administration to delivery, farrowing duration, birth interval, and placenta excretion time and also significantly increasing daytime delivery rates in sows. Kaeoket et al. (28) used *D*-cloprostenol sodium to induce parturition in sows at 0700 h on days 113 and 114 of gestation, resulting in the simultaneous delivery of offspring approximately 25 h after administration (at 0800 h on the following day). In our experiment, the sows treated with *D*-cloprostenol sodium or *DL*-cloprostenol sodium showed synchronized parturition approximately 23 h after initiation of administration (at 1,000 h on the next day). This finding challenges the previously held perception that starting treatment with PGF_2α_ drugs at 0700 h on the day prior to expected delivery may be inappropriate.

Farrowing duration is negatively correlated with colostrum yield; each additional minute of farrowing duration reduces colostrum yield by 2.2 g (2). The delivery interval is defined as the farrowing duration divided by the total number of piglets born minus one, and the cumulative birth interval is a significant risk factor affecting piglet colostrum consumption (29). Although oxytocin can improve the progress of parturition (30), prevent postpartum vaginal bleeding (31), and promote rapid milk discharge in lactating sows (32), in the long run, it may lead to lactation disorders and reduce the birth vitality of piglets (18, 30). Even when oxytocin analogs and *DL*-cloprostenol sodium are employed to induce parturition, both the colostrum yield of sows and the colostrum intake of piglets are notably lower than those with *DL*-cloprostenol sodium alone or natural delivery (4), which suggests that PGF_2α_ analogs may appear to improve colostrum yield.

Our results demonstrate that *D*-cloprostenol sodium and *DL*-cloprostenol sodium can significantly reduce the rates of Stillborn Type II in MS from 5.76% to 1.74–3.13%, which indicates that inducing parturients with cloprostenol sodium is able to reduce fetal mortality within the sow birth canal during deliver and also suggests that it can effectively shorten farrowing duration. Previous studies have shown that the induction of parturition using PGF_2α_ and its analogs can reduce stillbirth rates by 28% (18). Additionally, our results align with previous studies that have reported stillbirth rates of 3–8% for piglets born naturally (33). Our calculated rate is 8.73%, slightly exceeding the range, which is the sum of 5.76% (Stillborn Type II) and 2.97% (Stillborn Type I). The stillbirth rates of piglets account for 27% of deaths pre-weaning (34). In another investigation, parturition was induced in 77 sows through the injection of 175 μg *DL*-cloprostenol sodium at 0700 h on day 114 of pregnancy, resulting in a total stillbirth rate of 5.3% (53/1004), with Stillborn Type II accounting for 79.2% (42/53) (35). Although Nguyen et al.’s study lacked a blank control group, we observed that the Stillborn Type II in the group with natural farrowing was significantly higher than those in the group treated with *DL*-cloprostenol sodium in our experiment. This finding infers that the proportion of Stillborn Type II during natural farrowing was much higher than 79.2%. Therefore, inducing parturients can effectively decrease the incidence of stillbirth, thereby enhancing animal welfare.

### 4.2 Characteristics of sows of lactation and weaning characteristics of piglets

Lower back fat thickness at the end of pregnancy may potentially hinder breast development, while thickness in the week before delivery is positively correlated with colostrum yield and lactation duration (1, 36). In our experiment, to minimize the effect of back fat thickness in late pregnancy, the average back fat thickness of each subgroup was controlled at approximately 17 mm. Additionally, in the GLM procedure analysis of weaning litter weight and other parameters, we incorporated pre-delivery back fat as a covariate. Data show that the difference among groups was less than 2 mm. The back fat thickness of each group was approximately 15 mm on day 20 of lactation, with a decrease of approximately 2 mm compared to late pregnancy. In our experiment, multiple doses of *D*-cloprostenol sodium or *DL*-cloprostenol sodium did not affect the loss of back fat thickness in lactating sows. The research showed that the loss of 2.5–4 mm in thickness during lactation does not impair the reproductive capacity of sows in the following parity as these sows can regain the lost thickness prior to the next AI (37).

Insufficient feed intake by lactating sows is associated with increased mortality rates of pre-weaning piglets and prolonged WEI of sows in hot areas (26). In our experiment, the daily feed intake of sows exhibited a growth trend with an increased frequency, and as the injection frequency increased, both the number and weight of weaned piglets gradually increased. Increasing feed intake enhances the nutritional level of lactating sows, leading to an increase in the milk yield of sows and higher weaning litter weight of piglets (26). Additionally, the absorbed nutrients are primarily utilized by the mammary glands in sows during lactation (38), and approximately 70% of the total energy requirement and 90% of the absorbed amino acids are used for milk production and mammary gland development (39, 40). Therefore, it is speculated that cloprostenol sodium may improve lactation performance and mammary gland function by regulating the central nervous system in lactating sows.

In our experiment, sows injected with quartic doses of *D*-cloprostenol sodium observed a significant increase in the weaning litter weight by 24.98% and an 18.88% increase in the ADWG of the piglets. Despite an approximate 18% increase in the ADWG observed with *DL*-cloprostenol sodium, its impact on weaning litter weight was only 18.53%, which may be due to a slightly lower number of weaned piglets, as evidenced by the data. Approximately 4 g of milk are required for each gram of live piglet weight gain, which has not changed significantly over the last 30 years (41, 42). The algorithm for milk yield, as described by Lawlor et al. (17), indicates that the milk yield can be increased by 30.30% with quartic injections of *D*-cloprostenol sodium and by approximately 25% with repeated quartic injections of *DL*-cloprostenol sodium. Nonetheless, without considering optical isomers, repeating three injections of cloprostenol sodium only increased milk production by approximately 20%, which is lower than the enhancement achieved with four injections. Multivariable analyses conducted by Quesnel et al. (43) confirmed that colostrum intake was the predominant factor influencing piglet survival within 3 days after parturition, as well as pre-weaning survival, growth, and development. Administering dinoprost multiple times postpartum can increase the milk yield of sows and the colostrum intake of piglets, leading to increased litter weight of weaned piglets and improved growth performance of piglets before weaning (44). The milk yield of artificially lactating non-pregnant sows can be significantly increased 48 h and 108 h after the administration of PGF_2α_ (45). Notably, the chemical structure and half-life of cloprostenol sodium and dinoprost differ; cloprostenol sodium is more stable and has a longer half-life than dinoprost. Another study showed that *DL*-cloprostenol sodium-induced parturition tended to increase the colostrum yield of primiparous sows (4). Colostrum yield was not detected in this experiment. Nonetheless, our findings also demonstrated that the administration of *D*-cloprostenol sodium 24 h before delivery led to a significant increase in weaning weight and milk production within 21 days of lactation, while *DL*-cloprostenol sodium did not. These findings indicate that *D*-cloprostenol sodium may be more effective at increasing colostrum production.

Overall, the combination of inducing parturition before delivery and injecting multiple doses of cloprostenol sodium during the first 2 weeks of lactation can significantly enhance the growth of piglets and milk yield of sows. Among them, the administering of quartic injections (24 h prenatal; 3 h, 5 d, and 10 d postpartum) significantly improved the growth rate of piglets, with *D*-cloprostenol sodium proving to be the most effective.

### 4.3 Milk composition and lactation-related hormones levels in sows

Although there was no significant difference in lactose, milk fat, and milk protein between the sows treated with cloprostenol sodium and untreated sows, it was evident that four injections of two different types of cloprostenol sodium could lead to an increasing trend in milk protein and milk fat content during the middle and later stage of lactation. Other studies have shown that the content of lactose, fat, and protein in sows’ milk hardly varies greatly throughout lactation (46). These findings may also be related to the potential of multiple cloprostenol sodium in enhancing piglets’ weaning litter weight and ADWG.

Hormones such as PRL, E_2_, and P_4_ are involved in regulating animal estrus and are closely related to mammary gland development as well as the initiation and maintenance of lactation (40, 47). In this study, it was observed that multiple injections of cloprostenol sodium improved the growth performance of piglets, which may also be attributed to lactation-related hormones. PRL is the most important hormone involved in lactation in sows. Induced parturition with *D*-cloprostenol sodium can significantly increase the PRL levels in prenatal sows. PRL promotes the synthesis of lactose, milk fat, and milk protein by activating the JAK2-STAT5 and PI3K-AKT1-mTOR pathways through the PRL receptor. P_4_ is also essential for mammary gland development during puberty and pregnancy (48). Sow milk originates from blood circulation, but elevated serum P_4_ levels can affect the milk quality of sows and increase piglets’ diarrhea rates within 7 days (2). A sow that has just finished giving birth with high P_4_ levels would result in less weight gain or even weight loss on their first day. For some sows, P_4_ levels remained high even at 48 h after delivery, resulting in the average daily gain (ADG) of these piglets being below the normal level within 3 days of birth (49). The synthesis and secretion estrogen are influenced by the hypothalamic–pituitary axis. E_2_ stimulates the extension of mammary ducts, proliferation of breast epithelial cells, and lactation (50, 51). High levels of P_4_ before delivery inhibit the stimulatory effect of E_2_ on PRL (52). In the experiment, multiple postpartum injections had no effect on E_2_ and P_4_ levels.

Sows undergo significant metabolic and physiological changes during childbirth within a short period, and they face heat stress throughout the entire lactation period. High temperatures and intense sucking stimulation in piglets both increase COR levels in sows, resulting in immunosuppression (53). In our experiment, the sows treated with double cloprostenol sodium showed reduced COR levels in the first postpartum period. Previous studies have shown that COR can serve as a marker of stress during lactation and has a positive effect on sow lactation (54). Martinez-Miro et al. (54) revealed that high concentrations of COR in sow saliva were associated with increased piglet mortality within 3 days. When the concentration of COR in sow saliva is low, the growth rate of piglets increases during lactation (55). In our experiment, the administration of *D*-chloroprostol sodium did not effectively reduce the levels of COR in the late and middle stages of lactation. However, there was no negative impact on the ADWG of piglets.

### 4.4 Reproductive performance of weaned sows

Injecting 75 μg *D*-cloprostenol sodium before or after farrowing shortened the WEI and WSI in weaned sows by approximately 0.5 days, which can be attributed to the effective dissolution of corpus luteum of pregnancy by *D*-cloprostenol sodium. Recently, findings indicate that *DL*-cloprostenol sodium can dissolve the corpus luteum more effectively and reduce P_4_ levels more than dinoprostaglandin F_2α_ (56, 57). *D*-cloprostenol sodium is the dextral enantiomer of *DL*-cloprostenol sodium, which serves as the active component. In our experiments, the progesterone level-lowering effects were found to be equivalent when administering 75 μg of *D*-cloprostenol sodium and 200 μg of *DL*-cloprostenol sodium. Administration of PGF_2α_ analogs can induce the expression of endometrial growth factors, promoting endometrial repair and accelerating the elimination of postpartum placenta and other foreign bodies, thereby reducing the occurrence of uterine inflammation (58). Notably, the utilization of quartic dose of *D*-cloprostenol sodium showed an increase in conception rates by approximately 18 percentage points for the weaned sows. Thus, the multiple administration of cloprostenol sodium during lactation effectively improves the utilization rate of weaned sows.

This study represents the first demonstration that administration of cloprostenol sodium during lactation can improve the lactation performance of sows, which is very important for the health and welfare of both the sow and the piglets. We acknowledge the small sample size of blood and colostrum samples and recognize that the results could have been more robust with a larger number of samples. We also acknowledge the administration of postpartum antimicrobials to all sows; however, this practice lacks prudence. Antimicrobials should only be used in animals that are diseased, and prophylactic usage should be avoided. The proper use of antimicrobials should be considered in future studies. Moreover, we did not assess colostrum yield or immunological parameters of milk or investigate the effect of cloprostenol sodium on udder development in MS during lactation.

## 5 Conclusion

In conclusion, the administration of a single dose of *D*-cloprostenol sodium and *DL*-cloprostenol sodium in the prenatal 24 h can significantly shorten delivery process and reduce the rates of stillborn piglets type II in MS. The milk yield of sows can be significantly increased by inducing delivery with cloprostenol sodium, especially 75 μg *D*-cloprostenol sodium. Multiple postpartum administrations of cloprostenol sodium can significantly improve weaning litter weight, milk yield, average daily feed intake, and speed of weight gain in piglets. Furthermore, the sows treated with *D*-cloprostenol sodium exhibited enhanced PRL levels. In total, quartic doses of *D*-cloprostenol sodium are administered for 24 h prior to delivery and at 3 h, 5 d, and 10 d postpartum, yielding the optimal reproductive performance in sows. However, further investigations are needed to confirm the underlying molecular mechanisms for these observed effects.

## Data availability statement

The original contributions presented in the study are included in the article/supplementary material, further inquiries can be directed to the corresponding authors.

## Ethics statement

All procedures followed the guidelines for the ethical treatment of animals and were approved by the Animal Ethics Committee of the South China Agricultural University (Approval number SCAU#0025). The studies were conducted in accordance with the local legislation and institutional requirements. Written informed consent was obtained from the owners for the participation of their animals in this study.

## Author contributions

XuZ: Conceptualization, Data curation, Investigation, Writing – original draft. XiZ: Data curation, Writing – original draft, Formal analysis. YZ: Investigation, Resources, Validation, Writing – original draft. JL: Investigation, Resources, Validation, Writing – original draft. SL: Data curation, Writing – original draft. SiZ: Data curation, Writing – original draft. LL: Supervision, Writing – original draft. LM: Validation, Writing – original draft. HW: Funding acquisition, Writing – review & editing. ShZ: Funding acquisition, Methodology, Project administration, Writing – review & editing.
